# Urgency and necessity of Epstein-Barr virus prophylactic vaccines

**DOI:** 10.1038/s41541-022-00587-6

**Published:** 2022-12-09

**Authors:** Ling Zhong, Claude Krummenacher, Wanlin Zhang, Junping Hong, Qisheng Feng, Yixin Chen, Qinjian Zhao, Mu-Sheng Zeng, Yi-Xin Zeng, Miao Xu, Xiao Zhang

**Affiliations:** 1 State Key Laboratory of Oncology in South China, Collaborative Innovation Center for Cancer Medicine, Department of Experimental Research, Sun Yat-sen University Cancer Center, Sun Yat-sen University, Guangzhou, Guangdong PR China; 2 Department of Biological and Biomedical Sciences, Rowan University, Glassboro, NJ USA; 3 State Key Laboratory of Molecular Vaccinology and Molecular Diagnostics, National Institute of Diagnostics and Vaccine Development in Infectious Diseases, School of Public Health, Xiamen University, Xiamen, Fujian PR China; 4 College of Pharmacy, Chongqing Medical University, Chongqing, PR China

**Keywords:** Vaccines, Virology

## Abstract

Epstein-Barr virus (EBV), a γ-herpesvirus, is the first identified oncogenic virus, which establishes permanent infection in humans. EBV causes infectious mononucleosis and is also tightly linked to many malignant diseases. Various vaccine formulations underwent testing in different animals or in humans. However, none of them was able to prevent EBV infection and no vaccine has been approved to date. Current efforts focus on antigen selection, combination, and design to improve the efficacy of vaccines. EBV glycoproteins such as gH/gL, gp42, and gB show excellent immunogenicity in preclinical studies compared to the previously favored gp350 antigen. Combinations of multiple EBV proteins in various vaccine designs become more attractive approaches considering the complex life cycle and complicated infection mechanisms of EBV. Besides, rationally designed vaccines such as virus-like particles (VLPs) and protein scaffold-based vaccines elicited more potent immune responses than soluble antigens. In addition, humanized mice, rabbits, as well as nonhuman primates that can be infected by EBV significantly aid vaccine development. Innovative vaccine design approaches, including polymer-based nanoparticles, the development of effective adjuvants, and antibody-guided vaccine design, will further enhance the immunogenicity of vaccine candidates. In this review, we will summarize (i) the disease burden caused by EBV and the necessity of developing an EBV vaccine; (ii) previous EBV vaccine studies and available animal models; (iii) future trends of EBV vaccines, including activation of cellular immune responses, novel immunogen design, heterologous prime-boost approach, induction of mucosal immunity, application of nanoparticle delivery system, and modern adjuvant development.

## Introduction

Epstein-Barr virus (EBV) is a γ-herpesvirus that contains a double-stranded DNA genome of approximately 172 kb and is the first identified human oncogenic virus^1,2^. The EBV particle has a typical three-layer structure: the outermost lipid envelope displaying multiple glycoproteins responsible for cell entry, the middle tegument containing 20–40 proteins, and the inner pseudo-icosahedral nucleocapsid surrounding the DNA genome. The complete atomic models of the EBV icosahedral capsid, dodecameric portal, and capsid-associated tegument complex were resolved recently^3,4^.

EBV infects more than 95% of humans and establishes a lifelong infection^5^. The target cells of EBV infection are B cells, epithelial cells, natural killer (NK)/T cells, and macrophages. In vitro, EBV infection leads to latent infection in B cells and a lytic infection in epithelial cells^6^. Although the mechanisms of EBV entry into B cells and epithelial cells are very distinct, the fusion triggering protein gH/gL and the fusion protein gB are involved in both processes^7^. EBV enters epithelial cells and B lymphoblastoid cells through fusion at the plasma membrane, while endocytosis is required for B lymphocyte infection^8,9^. To be specific, B cell entry is initiated by the most abundant membrane glycoprotein gp350/gp220 binding to the complement receptor-2 (CD21/CR2) or to CD35 (CR1)^10–12^. Following endocytosis into B lymphocytes^9,13^, binding of the gp42 C-terminal region of the heterotrimer gH/gL/gp42 to human leukocyte antigens class II (HLA-II), thereby causes the structure to become a “closed” state^14,15^. This conformational change is thought to allow gH/gL/gp42 complex to interact with gB to initialize fusion with the endosomal membrane^16^. The mechanism of entry into epithelial cells is less well-defined. The first step involves binding BMRF2 to integrins to lessen the distance between the virus and cell^17^. Then gH/gL binds to ephrin receptor A2 (EphA2) and this interaction is thought to allow gH/gL to induce gB initializing membrane fusion^18,19^. Besides EphA2, αvβ5/β6/β8-integrins may also contribute to this process, but their roles have not been clearly defined^20^. In addition, the interactions of gH/gL with nonmuscle myosin heavy chain IIA and gB with neuropilin-1 contribute to this process^21,22^.

The EBV life cycle is complex and involves the expression of approximately 80 viral proteins. The latent-lytic switch is a particularly significant event in the EBV life cycle, but its mechanism remains unknown. Only nine proteins that contribute to B cell transformation and tumorigenesis are expressed during latent infection. They include six EBV nuclear antigens (EBNA-1, -2, -3A, -3B, -3C, and -LP) and three latent membrane proteins (LMP-1, -2A, and -2B)^23,24^. Lytic infection involves numerous proteins, which can be divided into immediate early, early, and late proteins according to their expression during the different phases of viral replication^25^. Among these proteins, the immediate early proteins Zta (BZLF-1) and Rta (BRLF-1) act as triggers of the EBV lytic cycle^25^. Immediate early and early proteins control genome replication and expression of late proteins^25^. Many lytic gene products remain to be characterized as their functions are still unclear^25^.

Even though EBV infection is associated with different pathologies, prophylactic or therapeutic vaccines are not yet available. In this review, we will summarize (i) the disease burden caused by EBV and the necessity of developing an EBV vaccine; (ii) previous EBV vaccine studies, available animal models, and human clinical trials; (iii) future trends of EBV vaccine studies, including activation of cellular immune responses, novel immunogen design, heterologous prime-boost approach, induction of mucosal immunity, application of nanoparticle delivery system, and modern adjuvant development.

## Why do we need an EBV vaccine?

**Fig. 1:**
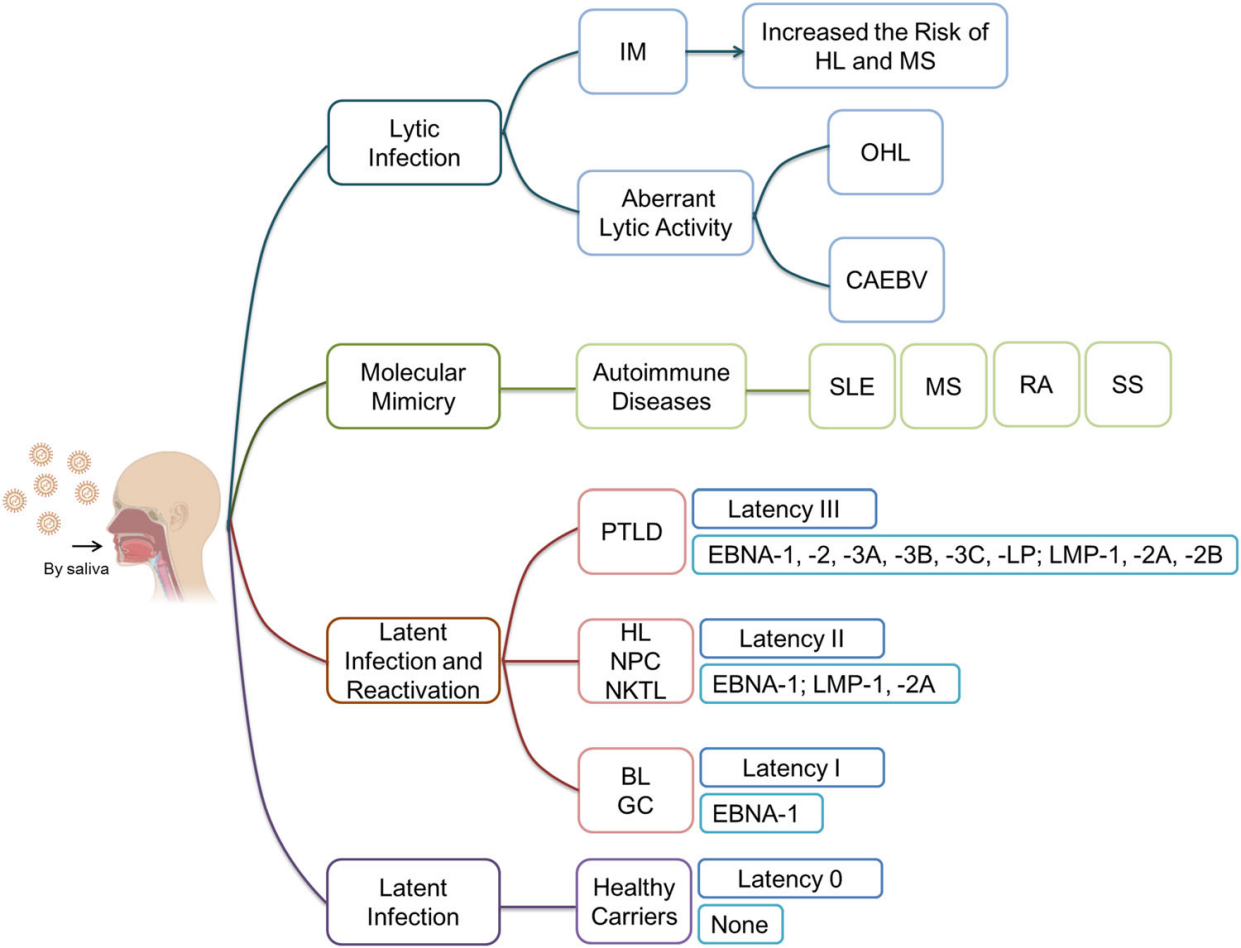
EBV-associated diseases and latency states. Primary EBV infection can cause infectious mononucleosis (IM), which also increases the risk of Hodgkin’s lymphoma (HL) and multiple sclerosis (MS). EBV aberrant lytic activity is associated with oral hairy leukoplakia (OHL), chronic active EBV disease (CAEBV). EBV infection and reactivation cause several autoimmune diseases, including rheumatoid arthritis (RA), Sjögren’s syndrome (SS), systemic lupus erythematosus (SLE), and MS through molecular mimicry. Besides, EBV is tightly associated with various lymphomas, including Burkitt’s lymphoma (BL), HL, natural killer/T-cell lymphoma (NKTL), as well as epithelial malignancies such as gastric carcinoma (GC) and nasopharyngeal carcinoma (NPC). The EBV latency state in HL, NKTL, and NPC is type II, while BL and GC display EBV latency type I. Besides these cancers, EBV latency is also associated to lymphoproliferative diseases in patients after stem cell transplantation or solid organ transplantation (PTLD). The figure was made from Biorender.com.

EBV is the pathogenic agent of diseases like infectious mononucleosis (IM), oral hairy leukoplakia (OHL), chronic active EBV disease (CAEBV), autoimmune diseases, and several malignancies (Fig. 1).

EBV primary lytic infection is associated with IM^26^. EBV primary infection in young children and adolescents remains asymptomatic, but young adults infected by EBV often develop IM, which also increases the risk of Hodgkin’s lymphoma (HL) and multiple sclerosis (MS)^27,28^. Boys with X-linked lymphoproliferative disease type I often develop severe IM after primary EBV infection, which may even become fatal^29,30^. Besides, EBV aberrant lytic activity is associated with OHL and CAEBV^31,32^.

Recently, as reported, EBV reactivation has been reported to enhance COVID-19 severity^33–36^. EBV infection and reactivation also contribute to the pathogenesis of several autoimmune diseases, including rheumatoid arthritis (RA), Sjögren’s syndrome (SS), systemic lupus erythematosus (SLE), and MS^37,38^. The EBV-specific antibodies and T cells generated during EBV infection and reactivation may react with autologous antigens and then causes autoimmune diseases^37,38^. Recently, a longitudinal study of a large cohort of subjects strongly supports the link between EBV infection and multiple sclerosis (MS)^39^. The risk of MS increased 32-fold after EBV infection^39^. Besides, antibodies against EBV nuclear antigen-1 (EBNA-1) induced during EBV infection cross-reacts with the central nervous system protein glial cell adhesion molecule and this molecular mimicry contributes to MS development^40^. However, molecular mimicry may be only one of the factors contributing to autoimmune disease pathogenesis.

EBV is tightly associated with various lymphomas, including Burkitt’s lymphoma (BL), HL, natural killer/T-cell lymphoma (NKTL), as well as epithelial malignancies such as gastric carcinoma (GC) and nasopharyngeal carcinoma (NPC)^41,42^. The oncogenic abilities of EBV latent proteins have been widely reported and EBV reactivation contributes to these malignancies^43,44^. EBV can adopt four latency states that are characterized by different protein expression patterns. Different malignancies are associated with different types of latent infections in the transformed cells (Fig. 1). The EBV latency state in HL, NKTL, and NPC is type II, while BL and GC display EBV latency type I. Beside these cancers, EBV latency is also associated with lymphoproliferative diseases in patients after stem cell transplantation or solid organ transplantation^45,46^.

Undoubtedly, EBV causes heavy global public health burdens. As reported, EBV caused 75,000 new cases/year of IM in the USA and 113,205, 105,554, 40,109, and 6318 new cases/year of GC, NPC, HL, and BL worldwide, respectively^41,47^. Remarkably, the geographical distribution of nasopharyngeal carcinoma is highly unbalanced and almost half of the new cases are in China, especially in South China^48^. In addition, the mortality of EBV-associated malignancies accounts for 1.8% of all cancer deaths^47,49–51^. Although NPC is highly sensitive to radiotherapy and chemotherapy, recurrence and metastasis are very common and linked to poor prognosis^52,53^. BL is a comparatively curable lymphoma, but the long-term survival rate is only 30–35% for relapsed or refractory BL in pediatric cases^54^. Hence, it is very necessary and urgent to develop an EBV prophylactic vaccine to prevent EBV infection and reduce the burden of all its associated diseases.

## Where are we now?

Due to the inherent tumorigenicity of EBV and the difficulty to achieve high virus production in cell culture, inactivated or attenuated vaccines are not available. From the 1970s onwards, multiple EBV vaccine studies encompassed subunit vaccines, epitope vaccines, DNA vaccines, nanoparticle-based vaccines, viral vector vaccines, virus-like-particles (VLPs), or dendritic cells (DC) vaccines (Table 1). The animal models that can be infected by EBV include humanized mice, rabbits, rhesus macaques (*Macaca mulatta*), common marmosets (*Callithrix jacchus*), cottontop tamarins (*Saguinus oedipus*), and owl monkeys (*Aotus trivirgatus*). In addition, five human clinical trials were completed, but none of these vaccines successfully prevented EBV infection in humans.

**Table 1:** Different types of EBV vaccine studies.

Types	Antigen	Formulation	Ref.
Subunit vaccine	gp350	Subunit gp350 adjuvanted with Freund’s adjuvant or alum	^57,63^
		Subunit gp350 adjuvanted with Alum	^55^
		Subunit gp350 adjuvanted with GLA/SE	^62^
		Subunit gp350 adjuvanted with ISCOMs	^72^
		Subunit gp350 adjuvanted with SAF-1 or alum	^60^
		Subunit gp350 adjuvanted with SAF-1	^61,70^
		gp350 incorporated into liposomes	^58,59,71,74^
		Tetrameric and monomeric gp350^1–470^ + CpG and alum	^56^
		Fc-gp350 adjuvanted with CpG OND/Alum	^79^
	gB	Trimeric gB adjuvanted with CpG and alum	^84^
	gH/gL	Trimeric and monomeric gH/gL adjuvanted with CpG and alum	^84^
	/	Glycoprotein complex	^83^
	EBNA-1	αDEC-205-EBNA-1 adjuvanted with poly(I:C)	^95^
Epitope vaccine	gp350	CTL epitopes of gp350 and gH adjuvanted with TT and IFA	^67^
DC vaccine	BZLF-1	DCs transfected to express BZLF-1	^98^
DNA vaccine	gp350	pCDNA3.1 plasmid encoding gp350	^64^
	gp350	plasmid of tetrameric gp350^1–470^	^56^
mRNA vaccine	gH/gL/gp220/gp42	mRNA-1189 (NCT05164094)	/
Nanoparticle vaccine	gH/gL/gp42	ferritin-gH/gL/gp42 + SAS	^85^
	gH/gL	ferritin-gH/gL + SAS	^85^
		nanoparticle displaying 60 copies of gH/gL	^86^
	gp350	ferritin-gp350 + SAS	^65^
	gp350	LS- or I3-01- gp350 domain I/II/III adjuvanted with MF59	^66^
Virus-like particles (VLPs)	/	EBV-VLPs deleted EBNA-2, LMP-1, EBNA-3A, -B, -C and BZLF-1	^102^
	/	EBV-VLPs deleted BFLF-1/BFRF-1A or BBRF-1	^103^
	gp350/gB/gp42/gH/gL	NDV-VLPs-gp350, gB, gp42, gH and gL	^87^
	gH/gL/EBNA-1	NDV-VLPs-gH/gL-EBNA-1	^100^
	gB/LMP-2	NDV-VLPs- gB-LMP-2	^100^
	gp350	NDV-VLPs-gp350	^75^
		HBc149 displaying immunodominant epitopes of gp350	^78^
	EBNA-1	Immunogenic particles containing EBNA-1 + poly (I: C)	^104^
Viral vector vaccines	gp350	VV expressing gp350	^68,69,73^
		Adv expressing gp350	^77^
	gp350/gB/EBNA-2 or EBNA-3C	VV expressing gp350, gB, EBNA-2, or EBNA-3C	^99^
	EBNA-1	Adv expressing EBNA-1 and VV-EBNA-1	^96^

*GLA/SE* glucopyranosyl lipid A incorporated into a stable emulsion, *ISCOMs* immune-stimulating complexes, *SAF-1* Syntex adjuvant formulation, *Fc* crystallizable fragment, *αDEC-205-EBNA-1* C-terminus of EBNA-1 fused with DEC-205 (a human endocytic receptor), *CTL* cytotoxic T lymphocytes, *TT* tetanus toxoid, *IFA* incomplete Freund’s adjuvant, *DCs* dendritic cells, *SAS* sigma adjuvant system, *LS* lumazine synthase, *NDV* Newcastle disease virus, *HBc149* hepatitis B core antigen, *VV* vaccinia virus, *Adv* adenovirus.

### Vaccine candidates

Considering the complexity of the EBV life cycle, EBV glycoproteins, lytic proteins, and latent proteins are all potential immunogens in EBV vaccine design. It is worth noting that the oncogenic potential of latent proteins should be avoided through proper modification of their immunogenic forms. It is likely that combinations of antigens will induce a more protective immune response, but much needs to be done to define the optimal selections of antigens or their combinations.

#### Vaccines using lytic glycoproteins as immunogens

##### gp350

gp350 is the most abundant glycoprotein on the EBV envelope and most previous vaccine studies focused on this antigen^55–79^. The selection of an adjuvant is one of the pivotal parts to develop an effective subunit and several combinations have been tested in various models.

Vaccines comprising monomeric gp350 (mono-gp350-based vaccines) have been combined with various adjuvants, including alum^55,57,60,63^, glucopyranosyl lipid A incorporated into the stable emulsion (GLA/SE)^62^, Syntex adjuvant formulation (SAF-1)^60,61,70^, immune-stimulating complexes (ISCOMs)^72^, Freund’s adjuvant ^57,63^, and incomplete Freund’s adjuvant (IFA)^57,67^ (Table 1).

The immune response to monomeric gp350 has been influenced by these different adjuvants. Mono-gp350 adjuvanted with alum protected three out of five cottontop tamarins from lymphoma and reduced secretion of EBV DNA in common marmosets^55,57,60^. Additionally, mono-gp350 adjuvanted with alum induced more robust protective responses than Freund’s adjuvant and IFA in common marmosets^57^. In a different study, mono-gp350 adjuvanted with alum elicited the same antibody levels in a rabbit model compared with SAF-1^60^. Cottontop tamarins inoculated with mono-gp350 and SAF-1 were protected from lymphoma (two out of three were free of lymphoma^61^; four out of four were free of lymphoma^70^). Furthermore, vaccines incorporating mono-gp350 into glycoside Quil A-based ISCOMs required a lower antigen dose to protect four out of four cottontop tamarins from tumorigenesis after EBV challenge^72^. Besides, after inoculation of mono-gp350 with the Toll-like receptor 4 (TLR4) agonist GLA/SE, a gp350-specific T cell response was elicited, and anti-gp350 antibodies were detected for more than a year, indicating a durable immune response in vaccinated mice^62^. In addition, mono-gp350 adjuvanted with Freund’s adjuvant required less antigen dose and induced higher neutralizing titers than that adjuvanted with alum in rabbit^63^. Finally, sera from rabbits and owl monkeys inoculated with gp350 alone could mediate antibody-dependent cellular cytotoxicity (ADCC)^76^.

To sum up, although the various combinations with mono-gp350 were not systematically compared, they illustrate the critical role of adjuvants to address the requirements for lower gp350 antigen doses, less frequent inoculations, and durable immune responses. One study showed that levels of neutralizing antibodies do not reflect the protective effect of a vaccine in common marmosets^57^ while other studies emphasized the essential role of neutralizing antibodies at prevention tumor prevention in cottontop tamarins^60,61^. Among the adjuvants formulated with mono-gp350, SAF-1 seems to be better than alum to attain protection^61,70^. GLA/SE is beneficial to induce cellular immune responses^62^. Importantly, even with the applications of various adjuvants, these vaccines still need to be inoculated several times in order to elicit immune responses that protect animals from lymphoma. With the development of novel adjuvants, mono-gp350-based EBV vaccines may be successful in preventing EBV infection and associated diseases.

Sera of mice immunized with non-adjuvanted liposomes incorporating monomeric soluble gp350 (lipo-gp350) neutralize EBV infection in vitro^74^. However, multiple inoculations of lipo-gp350 adjuvanted with lipid A (fraction from *E.coli* lipopolysaccharide) induced high titers of neutralizing antibodies in mice and cottontop tamarins^71^. After 17 immunizations, cottontop tamarins vaccinated with lipo-gp350 were protected from lymphoma, while those immunized six times with lipo-gp350 still developed lymphoma^58,59^. Therefore, liposome delivery of gp350 combined with an efficient adjuvant may be another potential strategy to develop an effective EBV vaccine.

The use of multimeric gp350 has also been explored because of its higher immunogenicity compared to monomers. Mice immunized with tetrameric gp350^1–470^ using alum and CpG oligonucleotides (CpG ODN) as adjuvants elicited much higher anti-gp350 antibody and specific CD4^+^ T cell responses than mice immunized with monomeric gp350^56^. The enhanced immunogenicity may be due to enhanced B cell receptor (BCR) binding and signaling, vaccine uptake, or presentation and trapping by follicular dendritic cells. The first step of B cell activation is BCR recognition and cross-linking, thus, multimeric antigens are more effective because they better mimic the natural arrangement of multiple copies of the antigen on the virion surface. In addition, a heterodimeric antigen consisting of a mouse IgG2a crystallizable fragment (Fc) fragment and gp350 induced higher neutralizing antibody titers in mice compared to monomeric gp350^79^.

As different approaches to gp350-based EBV vaccination, vaccinia virus and adenovirus were used as viral vectors to express gp350. The WR strain of vaccinia virus expressing gp350 (VV-gp350) induced humoral immune responses in rabbits, cottontop tamarins, and common marmosets^68,69,73^. It is remarkable that although no anti-gp350 antibodies and low levels of neutralizing antibodies were detected in cottontop tamarins inoculated with VV-gp350, three out of four animals were still free of lymphoma after an EBV challenge with a dose of 10^5.3^ lymphocytes-transforming doses that cause tumors in 100% of unvaccinated tamarins^73^. Similarly, all cottontop tamarins, which were vaccinated with a serotype 5 adenovirus expressing gp350 (Ad-gp350), were protected from lymphoma in vivo, even though their sera did not neutralize EBV in vitro^77^.

A DNA vaccine targeting antigen-presenting cells (APC) showed a good ability to elicit T-cell responses to gp350. Mice immunized with a recombinant pcDNA3.1 vector encoding gp350 induced not only gp350-specific antibodies but also cellular immune responses^64^. In a different study, sera from mice immunized with a plasmid expressing a gp350^1–470^ tetramer delivered with the PowderJect-XR-1 system showed higher antibody titers than those immunized with monomeric gp350^1–470^^56^. Those studies showed that nucleotide vaccines are attractive to improve immunogenicity and induce a stronger T-cell response that is crucial for killing EBV-infected cells. Note that only DNA vaccines have been studied and no data on RNA-based vaccines are currently available. mRNA vaccines for SARS-CoV-2 showed potent protective effects^80,81^. Recently, Moderna Inc. announced the initiation of a phase I study for its EBV mRNA vaccine mRNA-1189 (NCT05164094). The efficacy of such mRNA-based vaccines will likely influence the design of future EBV vaccines.

Other gp350-based vaccines include nanoparticle vaccines, epitope vaccines, and VLPs. Ferritin nanoparticles self-assemble to display 24 copies of gp350 (ferritin-gp350)^65^. These nanoparticles adjuvanted with Sigma Adjuvant System (SAS) elicited neutralizing antibodies in both mice and cynomolgus macaques^65^. Additionally, the immunized mice were protected from challenges with a recombinant vaccinia virus expressing gp350^65^. Nanoparticles of lumazine synthase (LS) or I3-01 displaying gp350 domain I/II/III induced higher titers of neutralizing antibodies than the monomeric form of gp350^66^.

Similarly, mice immunized with gp350 Cytotoxic T lymphocytes (CTL) epitopes combined with IFA and tetanus toxoid were also protected against the challenge of recombinant vaccinia virus expressing gp350^67^. The data highlight the importance of gp350 CTL epitopes and suggest that such epitopes are beneficial in the design of EBV vaccines.

Virus-like particles (VLPs) provide another attractive delivery system for EBV gp350 antigens. VLPs are multimeric self-assembled particles consisting of one or more structural proteins without a viral genome, which have no pathogenicity. Because their morphology and organization patterns are similar to natural viruses, VLPs can induce both cellular and humoral immune responses^82^. Chimeric VLPs based on the self-assembling hepatitis B capsid fragment hepatitis B core antigen (HBc149) were constructed to display three immunodominant epitopes of gp350^78^. In this system, these three peptides from the receptor binding domain of gp350 induced neutralizing antibodies in mice^78^. Interestingly, the humoral immune response was highly dependent on the sequential order in which these peptides were inserted in the HBc149 backbone^78^. VLPs based on Newcastle disease virus (NDV) capsid were constructed to display the ectodomain of gp350 (NDV-VLPs-gp350). These VLPs elicited a robust and durable neutralizing antibody response in mice^75^.

##### Other glycoproteins and combinations of glycoproteins

EBV entry into target cells is a well-organized and complex process. In addition to gp350, other glycoproteins are involved in virus entry and targeted by neutralizing antibodies. Central to the process of membrane fusion is the herpesvirus core fusion apparatus comprising gB trimers and gH/gL heterodimers^16^. Additionally, EBV B cell tropism is determined by the expression of gp42^16^. All these glycoproteins are potential antigens for vaccines aimed at neutralizing infection. Sera from rabbits inoculated with a mixture of glycoproteins prepared from the plasma membrane of EBV-positive P3HR-1 cells neutralized EBV in vitro^83^. Mice immunized with an epitope-based vaccine comprising gp85 (gH) and gp350 epitopes were protected from challenges with a recombinant vaccinia virus expressing gp85 or gp350^67^. Neutralizing titers of sera from rabbits immunized with trimeric or monomeric gH/gL, trimeric gB, and tetrameric gp350^1–470^ were much higher than those elicited by monomeric gp350^1–470^ (100-fold, 20-fold, 18-fold, and 4-fold higher, respectively)^84^. Ferritin nanoparticles containing gH/gL/gp42 elicited 2.5-fold higher neutralizing antibody levels against B cells infection and 250-fold higher neutralizing antibody titers against epithelial cells infection compared to ferritin-gp350^85^. In addition, nanoparticles displaying 60 copies of gH/gL instead of monomeric gH/gL induced neutralizing antibodies protected humanized mice from lethal EBV challenge^86^. Besides, a pentavalent vaccine based on NDV-VLPs containing EBV gp350, gB, gp42, gH, and gL was used with alum and monophosphoryl lipid A (MPLA) as adjuvants^87^. This cocktail induced neutralizing antibodies against infection of both B and epithelial cells in vitro^87^. Moreover, gH/gL/gp42 and gH/gL ferritin nanoparticles induced neutralizing antibodies in mice, ferrets, and nonhuman primates^88^. No immune competition was observed when combined with gp350D_123_ ferritin nanoparticles^88^. Besides, the passive transfer of antibodies purified from mice immunized with gH/gL/gp42 + gp350D_123_ or gH/gL + gp350D_123_ ferritin nanoparticles protected humanized mice from EBV-associated lymphoma^88^. These results clearly support the fact that gH/gL and gB are promising immunogen candidates. Recently, a clinical trial has been launched to evaluate an mRNA vaccine (mRNA-1189), which includes four mRNAs encoding gH, gL, gp42, and gp220 (NCT05164094). Overall, combining glycoprotein antigens is a promising approach for successful EBV vaccine development.

#### Vaccines using latent proteins and other lytic proteins as immunogens

Proteins that are not involved in virus entry should also be taken into consideration to develop effective vaccines for their expression in infected cells. These targets include other proteins of the lytic cycle as well as proteins expressed in various stages of latency.

In particular, EBNA-1 proved to be a robust immunogen. This antigen is expressed in almost all EBV-linked diseases and its role in maintaining the EBV genome in infected cells is a key factor in viral persistent infections^89^. EBNA-1 can be recognized by CD4^+^ T cells from almost all healthy carriers and EBNA-1-specific CD4^+^ and CD8^+^ T cells react with EBV-transformed B cells^90–94^. To use EBNA-1 as a vaccine, its C-terminus was fused with DEC-205 (a human endocytic receptor) and adjuvanted with poly (I:C)^95^. This vaccine candidate induced robust anti-EBNA-1 CD4^+^ and CD8^+^ T cell responses as well as anti-EBNA-1 IgM antibodies in humanized mice^95^. A heterologous prime-boost vaccination that combined a primary immunization with a recombinant adenovirus expressing EBNA-1 and a boost with a modified vaccinia virus Ankara (MVA) expressing EBNA-1 protected mice from EBNA-1 positive lymphoma after challenge^96^. Another nuclear antigen, EBNA-2 is one of the first viral proteins expressed during the initial stage of B cell immortalization^97^. EBV-infected B cells are recognized by EBNA-2-specific CD8^+^ T cells within 1-day post-infection and their proliferation can be prevented^97^.

BZLF-1 (Zta) has also been investigated as an immunogen. In a model of EBV-associated lymphoproliferative disease (LPD), survival rates of humanized mice significantly increased due to the specific CD8^+^ T cell response induced after inoculation of dendritic cells (DCs) transfected to express BZLF-1^98^. This result suggests that the BZLF-1-based vaccine could potentially prevent or delay EBV-associated diseases^98^.

#### Combinations of membrane glycoproteins, latent, and lytic proteins

The above studies indicate that proteins involved in virus entry, lytic infection, as well as latency, can contribute to an effective vaccine against EBV. It is, therefore, worth considering different combinations of latent and lytic proteins to develop a comprehensive cocktail vaccine. Toward that goal, a multivalent vaccine was devised by combining recombinant vaccinia viruses, each expressing gp350, gp110, EBNA-2, or EBNA-3C^99^. This cocktail induced CD4^+^ T cell responses and antibody responses in mice, indicating that the combination of different EBV proteins into a single dose produces the desired immune response^99^.

Heterologous VLP is another platform of choice to combine various antigens. NDV-VLPs-gH/gL-EBNA-1 and NDV-VLPs-gB-LMP-2 induced potent neutralizing antibodies as well as EBV-specific cellular responses in mice^100^. A different approach is to produce EBV-VLPs in non-transforming, virus-free packaging cell lines, using EBV genomes with deletions of some genes^101^. For instance, EBV-VLPs lacking major oncoproteins EBNA-2, LMP-1, EBNA-3A, -B and -C, and BZLF-1 can be produced in engineered 293-VII^+^ cells^102^. Such EBV-VLPs elicited potent humoral and cellular responses in mice^102^. Alternatively, EBV-VLPs with deletions of BFLF-1/BFRF-1A or of BBRF-1 induced a CD4^+^ T cell response^103^. The above EBV-VLPs usually contain many lytic proteins instead of latent proteins. Van Zyl et al.^104^ constructed more immunogenic particles by overexpressing EBNA-1 in producer cells. Humanized mice immunized with these EBNA-1-VLPs were successfully protected against EBV challenge^104^. However, except for latent proteins, BNRF1 and viral particles can also induce genetic instability and chromosome defects in infected cells^105,106^. Hence, safety evaluation of genetic instability and chromosome defects is needed for VLPs generated from non-transforming, virus-free packaging cell lines.

### Animal models

The lack of suitable animal models greatly hinders the research and development of EBV vaccines. Animal models which can be used to assess the protective effect of EBV vaccine candidates after EBV challenge, include humanized mice, rabbits, as well as nonhuman primates such as rhesus macaques, owl monkeys, cottontop tamarins, and common marmosets.

#### Humanized mice

Humanized mice are a novel model to investigate EBV infection and pathogenesis, study EBV-associated diseases as well as evaluate EBV vaccine candidates^107^. Humanized mice are based on immunodeficient mice, such as non-obese diabetic mice with *scid*, *RAG*, and/or IL-2 receptor γ chain mutations. These mice are transplanted with human CD34^+^ hematopoietic progenitor cells (HPCs) or peripheral blood mononuclear cells (PBMCs) from healthy donors. Infected cells in humanized mice express both latent and lytic EBV antigens after viral challenge^108^. Importantly, they can develop asymptomatic EBV infections, IM-like syndromes, or tumors depending on the EBV challenge dose, thus, they are useful models to study protection against EBV pathologies^108,109^. EBV-specific cellular immune responses are observed in humanized mice following EBV infection and the immune responses elicited by vaccines are similar to those of humans^110,111^. Furthermore, the innate immunity generated by reconstituted human NK cells also plays a significant role in the control of EBV lytic infections in this model^112^. However, humanized mice lack human epithelial cells, which are instrumental in the whole EBV infection cycle. Additionally, the development of “human” germinal centers and secondary lymphoid tissues is poor in this model^109,113^. Hence, the humoral immune responses cannot be reliably evaluated in the current humanized mice models. IgM antibody production against the viral capsid antigen BFRF-3 is detected in humanized mice^114^. Therefore, humanized mouse model is more suitable to evaluate the passive protective effect of antibodies purified from immunized mice, rabbits, or nonhuman primates. Studies using humanized mice to evaluate EBV vaccines are compiled in Table 2.

**Table 2:** Humanized mice models for EBV vaccines.

Year	Vaccine formulation and immunization route	Challenge strain	Results	Ref.
2008	αDEC-205-EBNA-1(aa400-641) + poly(I:C) i.p. twice at one month interval	None	EBNA-1 specific T cells and anti-EBNA-1 antibodies were detected	^95^
2015	rAd5F35/BZLF-1-transduced human DCs i.p. once or twice at a 2-week interval	None^A^	Prolonged survival to EBV-LPD	^98^
2018	immunogenic particles containing EBNA-1 + poly(I:C) i.p. twice at a 4-week interval	B95-8	Significant protection against EBV challenge	^104^
2022	Passive infusion of antibodies purified from mice immunized with gH/gL/gp42 + gp350D_123_ or gH/gL + gp350D_123_ ferritin nanoparticles 20 µg of mIgG per gram of mouse i.p. at day −1, day 0, and day 1	B95-8	Only one of six mice in each group received immune IgG had transient low-level viremia	^88^
2022	Passive infusion of antibodies purified from mice immunized with gH/gL 60 mer nanoparticle 500 mg of total IgG per mouse i.p. 48 h pre EBV challenge	B95-8	Purified antibodies from immunized mice protected humanized mice from lethal EBV challenge	^86^

*αDEC-205-EBNA-1* C-terminus of EBNA-1 fused with DEC-205 (a human endocytic receptor), *None* there is no challenge experiment. *i.p*. intraperitoneally, *DCs* dendritic cells. *EBV-LPD* EBV-associated lymphoproliferative diseases.

^A^humanized mice reconstituted with cells from an EBV-seropositive donor was used in this study.

#### Rabbits

Evidence showed that Japanese White rabbits can be persistently infected by EBV through intravenous inoculation since viral DNA and anti-EBV-VCA antibodies were both detected for 15 months^115^. Notably, persistent infections were also observed following infection of New Zealand White rabbits and Japanese White rabbits via the oral route, which is also the natural infection route in humans^116,117^. Furthermore, cells from New Zealand White rabbits infected intravenously proliferated in vivo following immunosuppression by cyclosporine A, which is reminiscent of observations in human post-transplantation lymphoproliferative disorder (PTLD) patients^118^. Together, these studies indicated that rabbit models are potential platforms for EBV vaccine evaluation.

#### Nonhuman primates

Rhesus lymphocryptovirus (rhLCV) is a homolog of EBV that only infects rhesus macaques and shares the same infectious features with EBV^119^. Experimental rhLCV infection in rhesus macaque causes either asymptomatic persistent latent infection or IM-like syndrome in immunocompetent macaques. However, in immunosuppressed macaques previously infected by simian immunodeficiency virus, rhLCV infection can lead to tumor formation^119–121^. Differences between rhLCV and EBV cannot be ignored, however, rhLCV vaccines and challenges performed in rhesus macaques can be considered as an indirect surrogate model to assess EBV vaccines^122^. Rhesus monkeys immunized with soluble rhLCV gp350 combined with alum as the adjuvant were protected against rhLCV oral challenge^123^. Interestingly, 72A1, a strong neutralizing monoclonal antibody targeting EBV gp350, protected rhesus macaques from oral challenge with a recombinant rhLCV carrying EBV gp350^124^. Such a chimeric virus may provide an interesting model to assess the in vivo protective effect of antibodies elicited by vaccine candidates.

Cottontop tamarins, common marmosets, and owl monkeys can be experimentally infected by EBV and recapitulate different aspects of human disease (Table 3). Cottontop tamarins are susceptible to experimental EBV infection and can develop malignant lymphomas after challenge with high doses of EBV^125,126^. Cleary and colleagues^127^ determined the 100% tumorigenesis dose of EBV strain B95-8 in cottontop tamarins and confirmed that tumors consisted of large-cell lymphomas with multiple copies of the EBV genome, which resembles the condition of PTLD patients. In addition, when cottontop tamarins recovered from tumors after the first challenge, cellular immune responses were observed after a second challenge, and these subjects remained healthy without any EBV-associated diseases^128^. Common marmosets can be infected by either the M81 strain (derived from an NPC patient) or the B95-8 strain (derived from an IM patient)^129–132^. The symptoms of infected common marmosets include lymphocytosis, the production of heterophile antibodies and the long-term production of EBV-specific antibodies are similar to those in humans^132^. After the EBV challenge, a persistent antibody response against EBV-VCA and early lytic proteins was observed^132^. However, antibodies against EBNA-1 were not detected and there were no viral antigens in the lymphocytes of infected animals, which differs from human cases^133^. In terms of pathologies, chronic infectious mononucleosis instead of LPD or lymphoma was observed in common marmosets. Owl monkeys also developed LPD after the experimental EBV challenge, and, interestingly, the EBV genome was found in a cell line established from an infected owl monkey^134,135^.

**Table 3:** Outcomes of Nonhuman primate after EBV infection reflect different aspects of human diseases.

Animal models	Reflection of human disease aspects
Cottontop tamarins	(a) EBV infection; (b) Malignant lymphomas after challenge with high doses of EBV; (c) Large-cell lymphomas with multiple copies of the EBV genome (resembles the condition of PTLD patients)
Common marmosets	(a) EBV infection; (b) Chronic infectious mononucleosis; (c) Lymphocytosis; (d) Production of heterophile antibodies; (e) long-term production of EBV-specific antibodies
Owl monkeys	(a) EBV infection; (b) LPD

*PTLD* post-transplantation lymphoproliferative disorder, *LPD* lymphoproliferative disease.

From 1980 to 2000, various EBV vaccines were assessed in these nonhuman primate models for efficacy (Table 4). Notably, sterilizing immunity was not achieved in any of these studies. Another limitation lies in the fact that experimental infection in nonhuman primate models is quite different from natural routes in humans. In some studies, data showed that there was no direct correlation between neutralizing antibody levels and vaccine protective effects, for some of the immunized animals with high neutralizing antibody levels still developed lymphoma after a 100% tumorigenesis virus challenge while those without high neutralizing titers free of lymphoma^59,73,77^. However, another study demonstrated that neutralizing antibodies is one of the key attributes of tumorigenesis prevention^58,60,61,70^. Therefore, additional factors may be involved to confer complete protection, such as cellular immune responses and ADCC.

**Table 4:** Nonhuman primate models for EBV vaccines.

Year	Animal	Vaccine formulation and immunization route	Challenge strain	Results	Ref.
1982	Owl monkey	Purified gp350 Two doses	None	Sera had neutralizing and ADCC effects	^76^
1984	Cottontop tamarins	Purified gp350 incorporated in liposomes + lipid A i.p. six times at a 3–9-week interval	None	Neutralizing antibodies were detected	^71^
1985	Cottontop tamarins	Purified gp350 incorporated in liposomes i.p. 17 times at a 2-week interval	B95-8	2/2 were free of lymphoma after 100% tumorigenesis dose challenge	^58^
1986	Cottontop tamarins	Purified gp350 incorporated into liposomes i.p. 6 times at a 2-week interval	B95-8	4/4 developed lymphoma after 100% tumorigenesis dose challenge	^59^
1988	Cottontop tamarins	Recombinant vaccinia viruses expressing gp350 (WR and Wyeth strains) i.d. 1 or 2 times at a 2-week interval	B95-8	Only the recombinant WR strain protected 3/4 of animals from lymphoma after a 100% tumorigenesis dose challenge	^73^
1988	Cottontop tamarins	Purified gp350 with ISCOMs s.c. three times at a 2-week interval	B95-8	4/4 were free of lymphoma after 100% tumorigenesis dose challenge	^72^
1989	Common marmosets	Purified gp350 with Freund’s or alum adjuvant i.m. 3 times at a 4-week interval	B95-8	Alum-adsorbed antigen-induced protection against virus challenge	^57^
1989	Cottontop tamarins	Purified gp350 with SAF-1 s.c. five times at a 2-week interval	B95-8	4/4 were free of lymphoma after 100% tumorigenesis dose challenge	^70^
1992	Cottontop tamarins	recombinant gp350 with BPV + SAF-1 adjuvant i.m. four times at a 10-day interval	B95-8	2/3 was free of lymphoma after 100% tumorigenesis dose challenge	^61^
1993	Cottontop tamarins	Recombinant adenovirus (serotype 5) expressing gp350 i.m. three times at 0-5-13 weeks	B95-8	4/4 were free of lymphoma after 100% tumorigenesis dose challenge	^77^
1994	Cottontop tamarins	recombinant gp350 with BPV + alum i.m. four times at a 4-week interval	B95-8	3/5 were free of lymphoma after 100% tumorigenesis dose challenge	^60^
1996	Common marmosets	Recombinant vaccinia virus expressing gp350 i.d. twice at a 5-week interval	M81	Replication of the challenge virus was decreased	^69^
1998	Common marmosets	recombinant gp350 with BPV + alum i.m. three times at a 4-week interval	M81	Replication of the challenge virus was decreased	^55^

*None* there is no challenge experiment, *ADCC* antibody-dependent cell-mediated cytotoxicity. *ISCOMs* immune stimulation complexes, *SAF-1* Syntex adjuvant formulation, *BPV* bovine papillomavirus expression vector, *i.p*. intraperitoneally, *i.d*. intradermally, *s.c*. subcutaneously, *i.m*. intramuscularly.

Finally, one should note that nonhuman primates are expensive and not necessarily amenable to large and preliminary studies of vaccine candidates. In addition, specific models for EBV, such as marmosets and owl monkeys, are rare and not readily accessible. Cottontop tamarins are not available since they are an endangered species.

### Clinical trials

From 1990 onwards, seven human clinical trials have been launched utilizing EBV gp350 or EBNA-3. For instance, Gu et al.^136^ utilized a live recombinant vaccinia virus (Tien Tan strain) expressing gp350 to immunize three groups of volunteers, including 11 adults (EBV seropositive and vaccinia seropositive), six juveniles (EBV seropositive and vaccinia seronegative), and 19 infants (EBV seronegative and vaccinia seronegative). In the adult group, antibody titers against EBV did not change after inoculation, while neutralizing antibody titers increased in young children and infants. Three out of nine infants still became naturally infected by EBV later. Meanwhile, ten out of ten control infants also became naturally infected. Moutschen and colleagues^137^ compared three vaccine formulations (recombinant gp350 alone, recombinant gp350 with alum, or recombinant gp350 with AS04) in seronegative and seropositive youths. All formulations were safe and well-tolerated. The formulation containing gp350 alone showed the weakest immunogenicity. Despite the detection of neutralizing antibodies and cellular immune responses, some subjects still became naturally infected. These observations are partly consistent with results obtained in common marmosets and cottontop tamarins, as discussed above. A phase II trial enrolled 181 seronegative young volunteers to test an EBV vaccine formulated with recombinant EBV gp350 and AS04 as an adjuvant^138^. Although anti-gp350 antibodies were detected over 18 months, this vaccine only prevented IM but not asymptomatic EBV infection. Another phase I trial recruited children with chronic kidney disease waiting for organ transplantation^139^. After inoculating two different doses (12.5 and 25 μg) of recombinant gp350 with alum, specific IgGs were found in all subjects. However, neutralizing antibodies were only detected in 1/4 of subjects who received the low dose and in 3/9 of subjects who received the high dose. Nevertheless, titers dropped quickly and vaccination did not affect the post-transplant immune condition of these children. Recently, a phase I clinical trial for a gp350-ferritin nanoparticle vaccine started to recruit subjects to evaluate vaccine safety and immunogenicity (NCT04645147). Another phase I clinical trial for an mRNA-based vaccine (mRNA-1189) containing four mRNA encoding gH, gL, gp42, and gp220 has been launched to evaluate to safety and tolerability of EBV mRNA vaccine in healthy adults ages 18 to 30 (NCT05164094).

Aside from glycoprotein-based vaccines, an EBNA-3 epitope-based vaccine was tested in 14 HLA B*0801-positive EBV-seronegative adults in a phase I trial^140^. This vaccine consisted of an EBNA-3 CD8^+^ epitope (FLRGRAYGL) combined with tetanus toxoid as CD4^+^ T cell helper and Montanide ISA 720 as the adjuvant. The vaccine proved to be safe and epitope-specific responses were observed, however, some immunized subjects seroconverted asymptomatically.

## What can we do?

Previous clinical trials failed to generate sterile immunity. However, all these efforts generated valuable information but also identified many barriers that need to be overcome to develop an effective EBV vaccine. Not only EBV has a complicated life cycle involving numerous proteins, but it also displays two distinct tropisms. Thus, the selection and design of the immunogen are still the key parts of EBV vaccine development. First of all, to improve current designs, T-cell epitopes should be taken into consideration. The balance of cellular and humoral immune responses is essential for an ideal vaccine, according to the previously successful herpesvirus vaccine against VZV^141^. Induction and evaluation of cellular immune responses will be necessary for novel EBV vaccine studies. Moreover, the rational optimization of immunogen combinations needs to be undertaken. Particle-based delivery systems and efficient modern adjuvants will help to improve immunogenicity, extend the duration of immune responses and reduce the inoculation doses. In addition, heterologous prime-boost immunization and induction of mucosal immunity are also promising. Finally, antibody-guided vaccine design will be worth trying according to the lessons learned from RSV and HIV vaccine studies^142^. We will briefly discuss these aspects.

### Immunogen design is the key part of vaccine development

An effective vaccine against a human herpesvirus is Shingrix^TM^ (GSK), which consists in glycoprotein E as the antigen and AS01_B_ as the adjuvant. This vaccine induces potent humoral and cellular immune responses, and successfully prevents shingles in the elderly^141,143^. The cellular immune response induced by Shingrix^TM^ plays a crucial role in disease prevention. It changes the paradigm that a robust humoral immune response could be sufficient for prophylactic vaccines against herpesviruses. Similarly, induction of T cell immune response is also essential and important for SARS-CoV-2 vaccine development^144^. Hence, T cell epitopes should be included for the balance of cellular and humoral immune response required from an effective EBV vaccine.

This realization is supported by data showing that immunized cottontop tamarins with high neutralizing antibodies titers can still develop lymphomas, indicating that induction of neutralizing antibodies alone is insufficient for disease prevention^59,73,77^. Cytotoxic and helper T-cell responses play a significant role in viral infection and disease prevention. Helper CD4^+^ T cells are essential for B cell and CD8^+^ T cell activation by providing secondary signals (e.g., CD28-B7) and cytokines secretion, respectively. In turn, cytokines secretion of CD4^+^ T cells determines the types of antibodies produced. Cytokines secreted by Th1 cells (e.g., IFN-γ) are helpful to produce IgG2a and IgG3 in mice or IgG1 in humans, while Th2 cytokines (e.g., IL-4) preferentially induce IgG1 and IgE in mice or IgG2 in humans^145^. CD8^+^ T and CD4^+^ T cells are especially important to kill infected cells^146,147^. Indeed, the CD8^+^ T cell response is the predominant response for eliminating EBV-infected cells during lytic and latent infections^148^. The important roles of T cells in controlling EBV infection warrant the inclusion of T cell epitopes in future EBV vaccines. Taylor et al. summarized the T cell epitopes of almost all EBV proteins, which provides a valuable resource to contribute to the design of future EBV vaccine candidates^149^.

Although there were strong justifications for using gp350, this limited antigen selection is likely one of the reasons for the failure to generate a sterile immunity in previous clinical trials. The EBV infection process is complicated and various glycoproteins (gp350, gH/gL, gp42, and gB for B cell infection and BMRF2, gH/gL, and gB for epithelial cell infection) are involved in EBV entry^17^. Anti-gHgL antibodies appear more effective since they neutralize ~75% of epithelial cell infection, while anti-gp350 antibodies neutralize ~57% of B cell infection^85^. Recent studies showed that gH/gL and gB elicited more potent neutralizing antibodies than monomeric gp350^1–470^, and ferritin-gH/gL/gp42 induced much higher neutralizing antibody titers compared to ferritin-gp350^84,85^. Additionally, passive transfer of antibodies induced by nanoparticles displaying 60 copies of gH/gL, gH/gL/gp42 + gp350D123, or by gH/gL + gp350D123 ferritin nanoparticles protected humanized mice from EBV-associated lymphoma^86,88^. Hence, gH/gL, gp42, and gB are effective and promising antigens for the development of prophylactic vaccines to be tested in clinical trials. Although the improved design may enhance gp350 immunogenicity, we believe that prophylactic vaccines with broader antigen spectra are more likely to be successful.

To broaden the spectrum of B cell and T cell epitopes, it is worth combining glycoproteins, latent and other lytic proteins into a single dose. Previous preclinical and clinical studies focusing on gp350 have been unsatisfactory, thus, other entry glycoproteins must be considered to enhance the production of neutralizing antibodies. In addition to well-characterized entry glycoproteins, the role of several membrane glycoproteins remains unknown, such as gp150 or gp78^150^, may eventually contribute to vaccine development. Besides, UV-inactivated EBV, used as a positive control in various experiments, elicited better neutralizing responses than NDV-based VLPs^75,87,100^. Hence, including more potential antigens in the appropriate vector will be crucial for the rational design of multivalent vaccines. However, it is important to note that not all formulations are effective and some even produce unanticipated effects. For example, sera from mice inoculated with both VLPs-gH/gL-EBNA-1 and VLPs-gB-LMP-2 led to increased EBV infection of epithelial cells rather than neutralizing infection as expected^100^. How to design the combination of EBV proteins is still a key issue.

### Heterologous prime-boost approaches

The vaccination prime-boost strategy is also important for vaccine efficiency. Heterologous prime-boost strategies were shown to be more immunogenic than homologous prime-boost approaches for HIV, herpes simplex virus type 2 (HSV-2), influenza, malaria, and tuberculosis^151^. Clinical trials for heterologous prime-boost SARS-CoV-2 vaccines are underway^152^. Examples of HIV vaccines have adopted “DNA prime-protein boost” strategies, which were able to induce both humoral and cellular immune responses^153–155^. A “DNA prime-viral vector boost” formulation against HIV has also been evaluated for its ability to induce cellular immune responses^156,157^. A “DNA prime-protein boost” approach for HSV-2 induced potent antibodies and both Th1 and Th2 immune responses^158^. An EBNA-1-based vaccine has been proven to be effective through an “EBNA-1-expressing adenovirus prime-EBNA-1-encoding MVA boost” strategy^96^. Experience from other enveloped viruses clearly indicates that a heterologous prime-boost strategy should be envisaged in the development of vaccines against EBV glycoproteins.

### Mucosal immunity

The mucosal immune response has to be considered an important aspect of the prevention of EBV infection since EBV primary infection occurs at oropharynx sites^6^. Vaccines designed to increase mucosal immunity are desirable to protect against EBV infection. Induction of IgA and tissue-resident T cells should be targeted and assessed in trials of EBV vaccines. Oral or intranasal immunizations are particularly effective at inducing mucosal immunity compared to injectable vaccines. Currently, the licensed mucosal vaccines comprise inactivated or attenuated viruses administrated orally or intranasally^159^. An inhaled SARS-CoV-2 vaccine (adenovirus type 5 vector), Convidecia Air^TM^, has been approved for Emergency Use Authorization as a booster dose^160^. A series of studies provide ample evidence supporting the use of intranasal and oral vaccines to trigger robust mucosal immune responses. Hence, oral or intranasal immunizations with the appropriate EBV antigens should be considered. For instance, an inactivated virus formulation could be developed as the main vaccine or as a booster after injectable vaccines.

### Particle-based vaccine is a promising field

Given that the immunogenicity of subunit and epitope vaccines has been usually insufficient, particles such as VLPs, protein scaffold nanoparticles, and polymer-based nanoparticles have been investigated. This approach is generally beneficial to enhance antigen immunogenicity since particles more closely mimic the characteristics of pathogens^161^.

VLPs are most similar to native viruses and have been used as immunogens in licensed HPV, HBV, and HEV vaccines^162^. VLPs are promising EBV vaccine candidates due to their high immunogenicity and their ability to induce potent humoral and cellular immune responses simultaneously, even without an adjuvant. In addition, VLPs can be designed to present various antigens or could be combined in vaccine formulations to expand the antigenic spectrum. As described above, protein scaffold-based nanoparticles, including ferritin, LS-, and I3-01 are promising to present EBV glycoproteins since they can induce much stronger immune responses than the soluble forms of the antigen^65,66,85^.

**Fig. 2:**
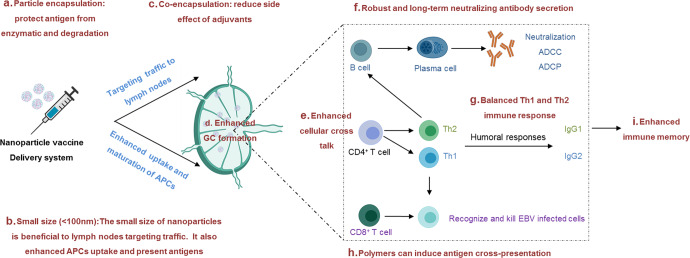
Nanoparticle delivery system. **a** Nanoparticle vaccine delivery systems can protect antigens from enzymatic degradation. **b** Depending on the size of the nanoparticles, they can be passively delivered to lymph nodes and enhance antigen-presenting cells (APCs) to take up and present antigens. **d** Hence, germinal center (GC) formation is enhanced. **c**, **e**–**h** Nanoparticle vaccine delivery systems can induce cellular and humoral immune responses simultaneously with minimum side effects. **i** Besides, nanoparticle vaccine delivery systems can improve the levels of antigen-specific memory T cells and B cells. The figure was made from Biorender.com.

Polymer-based nanoparticle delivery systems are highly versatile. They can be prepared with various materials such as chitosan, polyanhydrides, lactic acid, and poly (lactic-co-glycolic acid), for instance. Those nanoparticles can accumulate into lymph nodes and enhance antigen and adjuvant uptake by APCs depending on their sizes, surface charge, shapes, and hydrophobicity^163–166^. Additionally, polymers can protect antigens from degradation before uptake into target cells. Therefore, polymer-based nanoparticle vaccines can simultaneously induce cellular and humoral immune responses and reduce side effects (Fig. 2). Under ideal conditions, most nanoparticles are delivered to lymph nodes, where they effectively elicit robust humoral and cellular immune responses through enhanced uptake by APCs, promotion of BCR cross-linkage, as well as antigen cross-presentation (Fig. 2). Studies of nanoparticle vaccine against hand-foot-and-mouth disease or influenza, demonstrated the potential of polymer-based nanoparticles^167,168^. This approach exhibits the most advantages considered critical for the design of EBV vaccines.

### Application of modern adjuvants can enhance immune responses to EBV antigens

Adjuvant selection is an important component of vaccine formulation since it affects the number of immunization and doses of antigens needed to obtain a protective immune response^169,170^.

Many adjuvants have been approved (such as Alum, MF59, IFA, AS04, AS03, AS01b, CpG ODN, and IMQ) or are currently being tested in clinical trials (such as flagellin, Matrix-M, GLA-SE, ISCOMs, and AS02). A key role of traditional adjuvants is to build an antigen depot and improve the exposure time of the antigen. Modern adjuvants, such as agonizts of pattern recognition receptors (PRRs), concentrate on the activation of innate immunity. In the presence of PRR agonizts, APCs are activated to aid both cellular and humoral immune responses. Some TLR agonizts also interact directly with B cells and provide the co-stimulatory signals to activate T cells^171,172^. MPLA, an agonist of TLR4, induces Th1 and Th2 responses, while CpG ODN, an agonist of TLR9, is biased to induce a Th1-dominant response^173^. The combination of PRRs agonizts and nano delivery systems to form nano-adjuvant is also a promising approach. For example, the AS01_B_ adjuvant combines liposome as the delivery system and MPLA (TLR4 agonist) as the stimulating agent^174^. Shingrix^TM^, which effectively prevents shingles in the elderly, is an example of a vaccine using AS01_B_^141,143^. The application of the proper adjuvant or adjuvant system will greatly influence the success of subunit- and epitope-based vaccines against EBV.

### Antibody-guided vaccine design

Finally, antibody-guided vaccine design is an innovative way to develop vaccines^142^. Briefly, vaccine candidates are developed based on information obtained from the characterization of effectively neutralizing antibodies and their epitopes. The availability of neutralizing antibody libraries against corresponding antigens facilitates this approach. Relevant current examples of this strategy are found in vaccines targeting viral fusion proteins. The structure of human respiratory syncytial virus (RSV) pre-fusion F protein, together with analyses of neutralizing antibody complex, led to the design of stable pre-fusion F protein vaccines. This approach led to the development of an RSV vaccine candidate, which proved to elicit neutralizing antibodies^175,176^. This approach was also instrumental in designing RNA vaccines against SARS-CoV-2 expressing a stable pre-fusion form of the viral spike, which is the major target of most potent neutralizing antibodies^177,178^. These advances open exciting prospects for vaccines targeting EBV gB and available technologies must be used to determine the pre-fusion form of gB as the main goal of structure-based vaccine design.

Many EBV-specific monoclonal antibodies have been isolated and characterized. Thus, the tools are available to rationally design EBV vaccines according to neutralizing epitopes recognized by these antibodies^15,179–182^.

## Conclusion

EBV, as the first identified human oncogenic virus, causes a heavy health burden worldwide. It is imperative to develop an effective vaccine against EBV infection and EBV-associated diseases. Currently, none of the vaccine candidates are approved for clinical use, despite multiple attempts to develop an effective vaccine. Subunit vaccines, epitope vaccines, DNA vaccines, protein scaffold-based vaccines, viral vector vaccines, VLPs, and DC vaccines, all generated important information but generally failed to induce the required level of protection. Suitable animal models also need to be improved to study protection. Humanized mice, rabbits, rhesus macaques, and common marmosets are the most common animal models. However, each one of them has obvious limitations (as discussed above).

In human clinical trials, all vaccine candidates failed to prevent EBV infection. Induction of sterile immunity significantly correlates with a reduction of EBV-associated diseases. Hence, the ultimate goal of researchers is still to generate sterile immunity. Current efforts focus on antigen selection, combination, and design to improve the efficiency of vaccines. It is worth trying to develop a vaccine using new strategies for naïve pediatric populations to prevent the initial EBV infection. However, induction of sterile immunity may not be the only standard to evaluate the success of EBV vaccines. Preventing EBV-associated disease occurrence rather than EBV infection remains a valuable outcome when completely preventing EBV infection is not achieved. Vaccination with recombinant EBV gp350 adjuvanted with AS04 reduced the incidence of IM in seronegative subjects^138^. The incidence of HL and MS may be decreased accordingly^27,28^. Vaccination to reduce the incidence and severity of EBV-associated diseases is a valuable goal. Such goals were also established to evaluate the efficacy of SARS-CoV-2 vaccines in reducing symptomatic COVID-19^183–185^. Long-term clinical trials will be needed to assess the ability of the EBV vaccine to limit EBV-related diseases, in particular malignancies. Vaccination may also induce more potent cellular immune responses to control EBV reactivation in infected individuals^148^. Thus a vaccine that limits reactivation frequency and severity will have a valuable protective effect on infected individuals. This has been appreciated in the VZV vaccine Shingrix^TM^ (GSK), which successfully protects latently infected individuals from shingles^141,143^. EBV latent proteins and reactivation events are tightly associated with EBV-associated malignancies^43,44^. Hence, vaccination of infected populations has the potential to reduce EBV-associated diseases burdens. In particular, the population of south China, which is at high risk of developing nasopharyngeal carcinoma, will benefit greatly from such vaccination^186^.

Besides, delayed infection may occur due to the non-sterile vaccination. As discussed above, one consequence of a delayed infection after vaccination is that the infection will not cause diseases anymore, or reduce the severity of diseases. From the public health point of view, this would be a desirable outcome of vaccination greatly. However, whether the incidence of EBV-linked malignancies or autoimmune response can be reduced remains unclear. As observed in SARS-CoV-2 vaccination and repeated infection, the delayed infection of the Wuhan strain or other variants of concern also boosted immune responses^187^. Hence, it is possible that a delayed infection, whether successful or not, may also boost anti-EBV immune responses, thereby reinforcing the individual’s protection.

Induction of robust, long-term, and balanced humoral and cellular immune responses should remain the primary goal in the development of a protective EBV vaccine. The antigen spectrum, the immunogenicity of selected antigens, and the breadth of immune responses are the key issues to achieve this goal. Over the years, immunogen selection has changed from glycoproteins, especially gp350, to a more extensive range, including lytic and latent proteins. The identification and characterization of B and T cell epitopes of EBV protein help to further optimize immunogen design. Nanoparticle-based systems showed potential for vaccine development and novel adjuvant formulations are promising to increase immunogenicity. In addition, antibody-guided vaccine design provides a framework to improve EBV vaccine development based on the knowledge of EBV-neutralization acquired over many years.
